# Increased LDH5/LDH1 ratio in preoperative diagnosis of uterine sarcoma with inconclusive MRI and LDH total activity but suggestive CT scan: a case report

**DOI:** 10.1186/s12905-018-0662-5

**Published:** 2018-10-19

**Authors:** Antonio Mollo, Antonio Raffone, Antonio Travaglino, Annalisa Di Cello, Gabriele Saccone, Fulvio Zullo, Giuseppe De Placido

**Affiliations:** 1 Gynecology and Obstetrics Unit, Department of Neuroscience, Reproductive Sciences and Dentistry, School of Medicine, University of Naples Federico II, Via Sergio Pansini 5, 80131 Naples, Italy; 2 Anatomic Pathology Unit, Department of Advanced Biomedical Sciences, School of Medicine, University of Naples Federico II, Naples, Italy; 3 Gynecology and Obstetrics Unit, Department of Experimental and Clinical Medicine, University of Catanzaro Magna Graecia, Catanzaro, Italy

**Keywords:** Fibroid, LDH isoforms, Serum markers, Occult malignancy, Surgical approach, Choice of therapy, Leiomyoma, Menorrhagia, Minimally-invasive surgical treatments

## Abstract

**Background:**

Morcellation of undiagnosed uterine sarcoma is cause of abdominal/pelvic dissemination, residual tumor and recurrence. In the preoperative evaluation of suspect uterine masses, magnetic resonance imaging (MRI) and serum lactate dehydrogenase (LDH) total activity are referred to as the most effective tools, while computed tomography scan (CT) and LDH isoenzymes are less considered in literature.

**Case presentation:**

A 46 year old woman was admitted to our department with a large uterine mass. Ultrasonography, MRI and LDH total activity did not allow a diagnosis of malignancy, and the woman expressed the wish to avoid hysterectomy. In spite of this, we opted for a total abdominal hysterectomy instead of a laparoscopic myomectomy, due to an elevation of LDH5/LDH1 ratio and CT findings indicative of sarcoma. Histological examination revealed a high grade leiomyosarcoma, confirming our suspicion. Thus, we had avoided the risks linked to morcellation.

**Conclusions:**

Our experience suggests that LDH isoenzymes assessment may be relevant in preoperative diagnosis of uterine sarcoma. Further studies are necessary to determine its role in a diagnostic algorithm. We think it may be useful especially for patients with clinical or ultrasonographic suspicion of uterine sarcoma not confirmed by imaging techniques. Furthermore, the role of less considered imaging techniques, such as CT, should not be underestimated in challenging cases.

## Background

Uterine sarcoma is a rare malignant tumor. The incidence rates range from 0.35 to 1.53 of 100.000 among women in Europe. The most common subtype is leiomyosarcoma, which constitutes 60–70% of all uterine sarcomas. It occurs usually in post-menopausal women, with a median age of 55 years. The prognosis is very poor, with a 5-year survival rate of 41%. Even in stage I the recurrence rate is from 57 to 71% [1]. The prognosis is linked to the surgical radicality particularly in early stages, so the treatment of choice for sarcomas is *en bloc* resection or compartmental surgery constituted by abdominal hysterectomy, which correlates with better oncologic outcomes [2]. Therefore, given the widespread use of laparoscopic morcellation to remove benign uterine masses, preoperative identification of sarcoma is a crucial problem, as pointed out in a Food and Drug Administration safety communication published on April 17, 2014 [3]. In fact, in a variable percentage of cases (in a recent study 0.09–0.99%), histological examination of surgical morcellated specimen reveals an unexpected malignancy [4]. This leads to a worsening of prognosis, since several studies demonstrated that morcellation of sarcomas increases the risk of abdominal/pelvic dissemination, residual tumor and recurrence [1, 2, 4].

Despite the efforts to improve it, preoperative diagnosis of uterine sarcoma is still difficult and a validated diagnostic algorithm does not exist.

The clinical presentation is unspecific and the most common symptom is abnormal vaginal bleeding observed in about 53% of cases [5]. Several studies reported MRI as the most reliable imaging techniques [2, 4–7] and lactate dehydrogenase (LDH) as the most relevant serum marker [1, 2, 4, 6–9].

We report our experience about this issue, illustrating a case of leiomyosarcoma treated by total abdominal hysterectomy instead of a laparoscopic myomectomy because of characteristic LDH isoenzymes pattern supported by suggestive CT findings, although MRI and LDH total activity resulted to be inconclusive.

## Case presentation

A 46 year-old woman (gravida 2, para 2) with history of menometrorrhagia for 5–6 years due to a voluminous uterine fibroid was admitted to our institution (Department of Neuroscience, Reproductive Sciences and Dentistry, School of Medicine, University of Naples Federico II, Naples, Italy) with fever (temperature over 39 °C) and strong pelvic pain.

Transvaginal ultrasound (US) showed diffuse fibromatosis and two evident uterine masses: the first was 53 × 57 mm, submucous, in fundus-anterior wall; the other was 97 × 70 mm, subserous, in isthmus-posterior wall. Despite the size of the masses, no alarming features were observed.

In order to preserve pelvic stability, the woman expressed the wish to avoid total hysterectomy; therefore, a laparoscopic myomectomy was considered.

During hospitalization the patient showed an inflammatory state (elevation of fibrinogen and C-reactive protein) with intermittent fever (not exceeding 38 °C). Blood cultures were negative.

Several measurements of serum LDH total activity were performed, showing normal or only slightly increased values, with the highest peak of 304 U/l (reference range: 125–243).

**Fig. 1:**
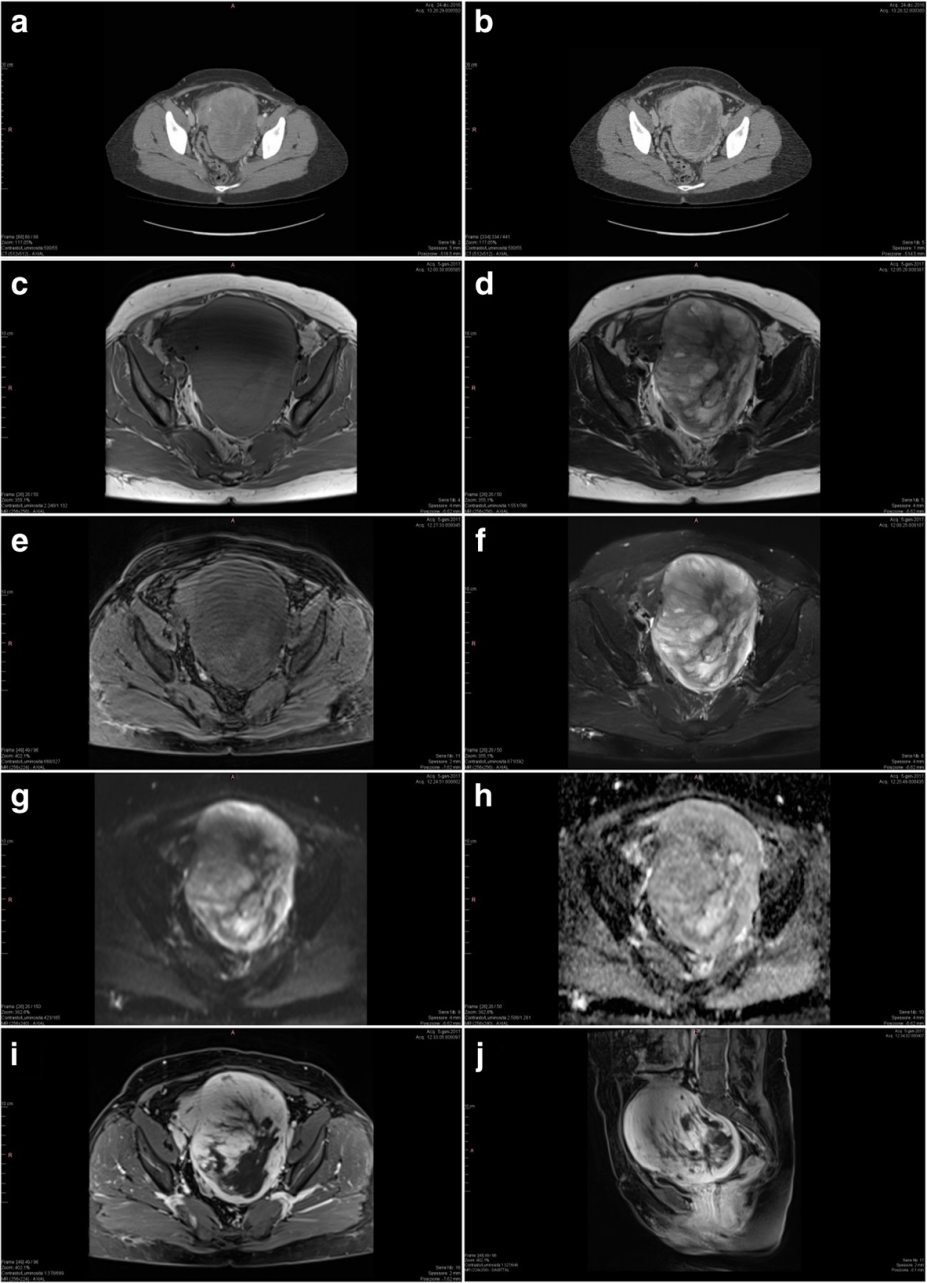
Imaging findings. Computed tomography scan without (**a**) and with contrast (**b**) showed the two uterine masses; the large one with eccentric areas of colliquative necrosis. On magnetic resonance imaging, the mass appeared capsulated and not infiltrative. **c** T1-weighted turbo spin echo (tse), transverse scanning plane (t); **d** T2-weighted tse; **e** T1-weighted tse fat suppression (fs), t; **f** T2-weighted tse fs, t; **g** Epi spair diffusion-weighted imaging (DWI), t; **h** Epi spair apparent diffusion coefficient maps (adc), t; **i** T1 volumetric interpolated breath-hold examination, volumetric interpolated brain examination (vibe) breath holding (bh), t; **j** T1 vibe bh, sagittal scanning plane (s)

Due to the clinical presentation, an abdominal CT with and without contrast was performed, showing increased uterine volume with two evident masses: the smaller one (4 × 3 cm) was subserous, on the fundus; the larger one (12 × 10 cm), voluminous and inhomogeneous, was para-uterine, on the left, with intraligamentary growth and eccentric areas of colliquative necrosis (Fig. 1). These features were suggestive of sarcomatous degeneration, so a MRI was recommended.

The abdomino-pelvic MRI was performed with and without contrast, confirming the presence of both masses (3.2 × 5 cm and 15x10x9 cm). However, the larger one appeared capsulated and non-infiltrative (Fig. 1). Characteristic MRI findings associated with sarcomas were not clear: enhanced signal intensity (SI) was absent in T1, and weak and inhomogeneous in T2. These findings were not enough to support the diagnosis of uterine sarcoma.

In order to support our suspicion of malignancy, electrophoresis of LDH isoenzymes was performed, showing the following results: LDH1 = 15.6% (reference range: 16.1–31.5%); LDH2 = 23.3% (29.2–41.6%); LDH3 = 21.0% (17.0–26.2%); LDH4 = 13.6% (5.9–12.3%); LDH5 = 26.5% (3.2–17.3%); LDH5/LDH1 ratio = 1.7 (normal value: < 1).

We found an elevation of muscle isoforms (4 and 5) accompanied by a decrease of the normally predominant heart isoforms (1 and 2), with an increased LDH5 to LDH1 ratio. In literature, this pattern has been presented as strongly associated with malignancy [9].

**Fig. 2:**
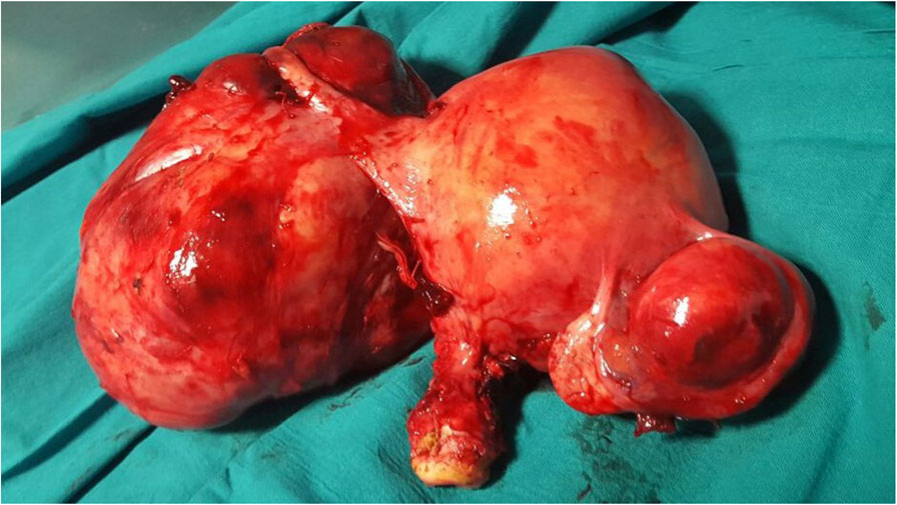
Macroscopic appearance of the surgical specimen

Given the characteristic LDH isoenzymes pattern, supported by the CT report, we discussed with the patient about the increased suspicion of malignancy and the necessity of a radical surgical treatment. So, we finally opted for a total abdominal hysterectomy instead of a laparoscopic myomectomy (initially preferred by the patient), in order to avoid risks linked to the morcellation of an occult malignancy (Fig. 2).

**Fig. 3:**
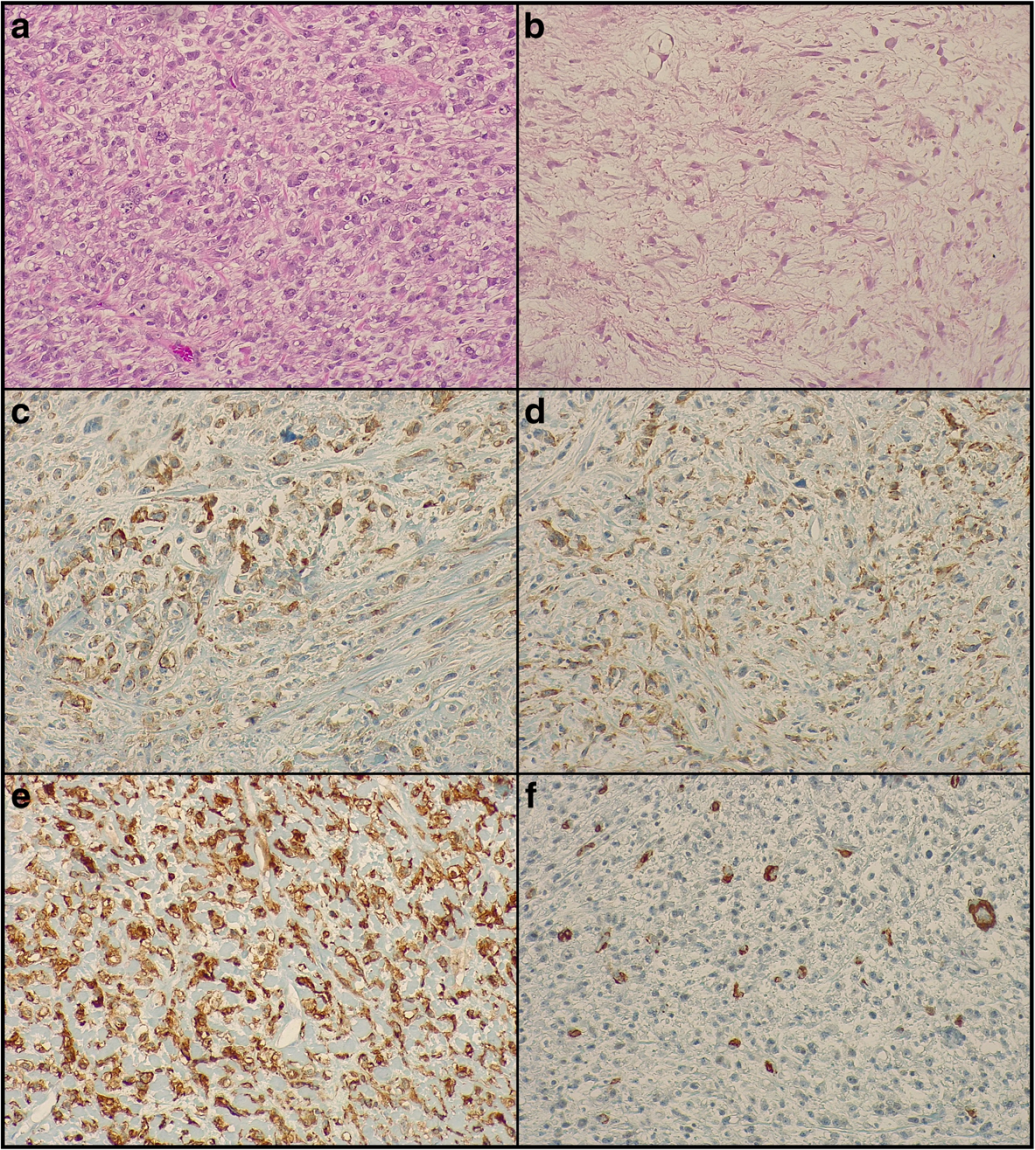
Histological features of the sarcoma (magnification 200x). The tumor showed hypercellular areas constituted by pleomorphic cells (**a**) alternating with hypocellular myxoid areas (**b**). The tumor was positive for muscle specific actin (**c**), caldesmon (**d**) and CD10 (**e**) and focally positive for smooth muscle actin (**f**)

Histological examination of the surgical sample showed a malignant mesenchymal proliferation constituted by hypercellular areas with epithelioid or pleomorphic cells (Fig. 3a) alternating with hypocellular myxoid areas (Fig. 3b). Necrosis and high mitotic index were observed. Immunohistochemistry showed positivity for caldesmon, muscle specific actin and CD10 and focal positivity for smooth muscle actin (Fig. 3c-f). The definitive diagnosis was of high grade leiomyosarcoma with myxoid changes, confirming our suspicion.

## Discussion and conclusions

Despite being expensive, MRI is the most studied tool in the preoperative diagnosis of uterine sarcomas. MRI findings associated with sarcomas are enhanced SI in T1 due to hypervascularization and inhomogeneous SI in T2 with areas of low-intensity due to necrosis and/or haemorrhage. More important appears to be an intermediate to high signal on diffusion weighted imaging (DWI) associated with a low apparent diffusion coefficient (ADC) [2, 4–7]. Though MRI showed positive and negative predictive value respectively up to 83.3% and 100% [7], problems about sensitivity (alarm features not ever present) and specificity (abnormal patterns exhibited by benign lesions) still persist [4, 5, 10].

CT often shows haemorrhage, necrosis and spread beyond the uterus as features associated with sarcomas, though its efficacy in differentiating them from myomas is lower than MRI [10] and is not supported by scientific data [2, 4].

Despite its marginal role in literature, in the case we presented CT was useful in supporting the suspicion of malignancy, even in presence of inconclusive MRI findings. In our view, although in literature MRI showed a greater reliability when compared with other imaging techniques, it has not yet reached the sensitivity and specificity needed to be the only decisive tool in the preoperative assessment of uterine masses. Anyway, MRI remains an indispensable diagnostic method when uterine sarcoma is suspected.

In several studies, LDH is reported as the most relevant serum marker in the preoperative assessment of suspect uterine masses [1, 2, 4, 6–9]. In this respect, Goto et al. proposed the association of increased LDH total activity with suggestive MRI findings, reporting both positive and negative predictive value up to 100% [7]. Nagai et al. included LDH total activity in a preoperative sarcoma score together with MRI findings (then excluded in a subsequent study), patient age and endometrial cytology, indicating a cut-off of 279 U/L and reporting positive and negative predictive value respectively of 92.3% and 94% [6, 8]. In spite of this, in our case LDH total activity showed normal values alternating with only two slightly elevated values (up to 274 U/L, still below the cut-off of 279 proposed by Nagai et al.).

Regarding the determination of LDH isoenzymes, a characteristic shift to a predominance of muscle isoforms (LDH4 and 5) with a decrease of the normally predominant heart types (LDH1 and 2) and increased LDH5 to LDH1 ratio has long since showed an association with malignancy [9]. To the best of our knowledge, the relevance of LDH5/LDH1 ratio with specific regard to uterine sarcoma was assessed only in one study including only 7 cases, with inconclusive results [11]. In our experience, the described pattern was decisive in the choice of surgical treatment and approach, leading us to finally perform an abdominal hysterectomy instead of a laparoscopic myomectomy.

Our experience suggests that LDH isoenzymes pattern may play a role in preoperative evaluation of suspect uterine mass, even with normal LDH total activity. However, further large and well-designed prospective studies are necessary to determine its actual reliability. If the significance of LDH5/1 ratio in uterine sarcoma will be confirmed, it may be integrated in a diagnostic algorithm and assessed in patients with clinical or ultrasonographic suspicion of uterine sarcoma not supported by imaging techniques.

However, as long as a validated and reliable diagnostic protocol does not exist, also the role of less considered imaging techniques, such as CT, should not be underestimated.

## Abbrevations

CT: Computed tomography scan
LDH: Lactate dehydrogenase
MRI: Magnetic resonance imaging
